# Optochemical control of G1 cell cycle by regulating CDK4/6 degradation

**DOI:** 10.1016/j.isci.2025.113304

**Published:** 2025-08-06

**Authors:** Tianyi Wang, Yaming Zhang, Yuwei Liu, Lichao Wang, Lijun Liu, Weiping Wang

**Affiliations:** 1 Department of Nasopharyngeal Carcinoma, Sun Yat-sen University Cancer Center, State Key Laboratory of Oncology in South China, Collaborative Innovation Center for Cancer Medicine, Guangdong Key Laboratory of Nasopharyngeal Carcinoma Diagnosis and Therapy, Guangdong Provincial Clinical Research Center for Cancer, Guangzhou 510060, China; 2 State Key Laboratory of Pharmaceutical Biotechnology, The University of Hong Kong, Hong Kong, China; 3 Department of Pharmacology and Pharmacy, Li Ka Shing Faculty of Medicine, The University of Hong Kong, Hong Kong, China; 4 Dr. Li Dak-Sum Research Centre, The University of Hong Kong, Hong Kong, China; 5 National Frontiers Science Center for Industrial Intelligence and Systems Optimization, Key Laboratory of Bioresource Research and Development of Liaoning Province, College of Life and Health Sciences, Northeastern University, Shenyang 110819, China

**Keywords:** Biochemistry, Cell biology, Cancer

## Abstract

Cancer progression is characterized by dysregulated G1/S phase transition mediated by CDK4/6-dependent Rb protein phosphorylation. Although CDK4/6 degraders show encouraging anti-tumor efficacy, it is highly desired to develop strategies to spatiotemporally control the release of active CDK4/6 degraders to further reduce adverse effects. In this study, we employ an optochemical strategy for CDK4/6 degradation by caging the CRBN ligand with a photoremovable protecting group. Light irradiation at 365 nm triggers photocleavage, thereby inducing CDK4/6 degradation via the ubiquitin-proteasome system. The resultant G1-phase arrest demonstrates spatial and temporal control over cell-cycle progression, reducing off-target effects of current therapies. This light-controlled system allows spatiotemporal CDK4/6 degradation and G1 cell-cycle arrest, providing a promising strategy to enhance specificity for cancer treatment and fundamental biological research.

## Highlights

• Opto-PROTAC shows efficient photocleavage under 365 nm light
• Opto-PROTAC achieves light-triggered CDK4/6 degradation
• FUCCI demonstrates that Opto-PROTAC can spatiotemporally induce G1 cell-cycle arrest

## Graphical abstract

**Figure:**
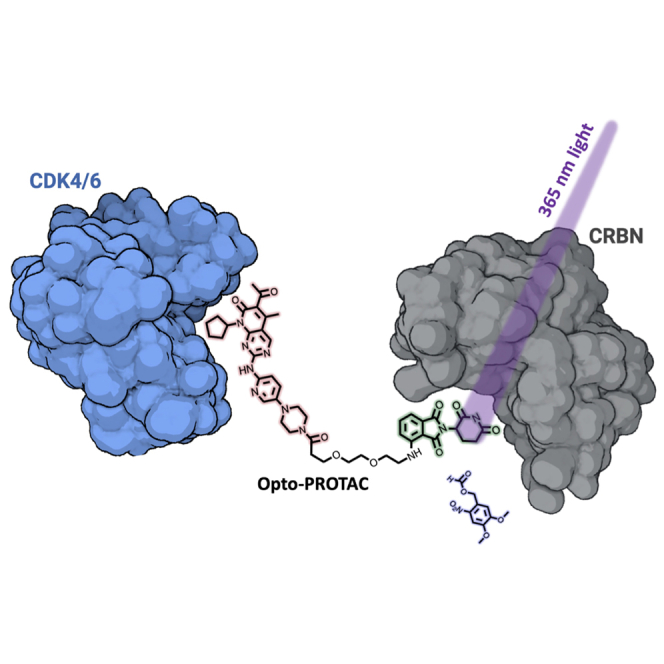

## Introduction

Cancer remains a leading cause of global mortality, driven largely by dysregulated cell-cycle dynamics. Cyclin-dependent kinases 4 and 6 (CDK4/6) play a central role in this process, which orchestrate G1/S-phase progression by phosphorylating the retinoblastoma (Rb) protein, thereby releasing E2F transcription factor to initiate DNA replication.^1^ In cancer, hyperactivation of CDK4/6 due to mutation, gene amplification, or mitogen-independent signaling often bypasses Rb-mediated cell-cycle checkpoints, enabling uncontrolled proliferation.^2^ As a result, CDK4/6 inhibitors, such as palbociclib, can be used to inhibit the function of these proteins, which can lead to G1 cell-cycle arrest. Yet, these inhibitors have to continuously occupy the binding sites of CDK4/6 for action to achieve a long-lasting therapeutic effect.^3^ Additionally, beyond the canonical kinase function in regulating Rb phosphorylation, CDK4/6 also exhibit other non-kinase functions critical to tumorigenesis, including regulation of cell motility by cytoskeletal remodeling, mitochondria activation by metabolic reprogramming, and immunomodulation by presenting tumor antigens and overexpressing PD-L1.^4^^,^^5^^,^^6^^,^^7^ Yet, palbociclib may not control the non-kinase functions of CDK4/6, as it mainly binds to ATP-binding sites and influences phosphorylation status without influencing protein expression levels.^8^^,^^9^

To overcome the aforementioned weaknesses, proteolysis targeting chimeras (PROTACs) are developed to mediate CDK4/6 degradation. These PROTACs are heterobifunctional constructs, containing a warhead targeting CDK4/6 proteins connected via a linker to a CRBN E3 ligase ligand, pomalidomide.^10^ Recruitment of E3 ligase to the proximity of CDK4/6 proteins leads to their ubiquitination andsubsequent proteasomal degradation.^10^^,^^11^^,^^12^^,^^13^^,^^14^ By degrading CDK4/6 rather than inhibiting kinase activity, PROTACs can prolong the degradation effects and suppress both kinase and non-kinase functions.^8^^,^^9^^,^^15^ However, the introduction of ligand for E3 ligase may lead to uncontrolled protein degradation or influence proteasome function significantly in normal cells. Moreover, recent studies found that the functions of CDK4/6 were highly associated with their sub-cellular location. For example, Dawn et al. found CDK6 was predominantly localized in the cytoplasmic edges to regulate cytoskeleton remodeling, while the nuclear fraction of CDK6 was active as Rb kinase.^16^ Yet, it is not thoroughly understood whether other non-kinase functions of CDK4/6, such as the regulation of mitochondria activity or anti-cancer immunity, are location-related. Defining the sub-cellular locations of CDK4/6 that are associated with these non-canonical functions could facilitate deeper understanding of related biological processes and mechanisms. Therefore, it is highly desired to develop chemical tools to spatiotemporally control CDK4/6 degradation and improve the specificity of CDK4/6 PROTAC for cell-cycle arrest.

In recent years, significant progress has been achieved with the introduction of photo-pharmacological concepts, enabling spatiotemporal manipulation of small-molecule drug bioactivities.^17^^,^^18^ Light-responsive molecules are developed by conjugating the active moiety of parent molecules with a photoremovable group, which can be cleaved upon light irradiation to release the active parent molecules, thereby triggering corresponding bioactivities.^19^^,^^20^ As an example, a proteasome inhibitor, MG-132, was photocaged directly at the reactive aldehyde function with a photoremovable group. After light irradiation, the photoremovable group was cleaved and MG-132 was activated, which blocked the UPS-dependent protein degradation and led to metaphase cell-cycle arrest.^21^ However, as proteasome inhibitors block the degradation of various proteins and influence multiple biological processes, including endoplasmic reticulum stress and reactive oxygen species (ROS)-mediated DNA damage,^22^ they may not be ideal for specifically investigating cell cycle-related bioactivities.

By combining PROTAC technology and photopharmacology, optochemical control of protein degradation can also be achieved. For example, BRD4 PROTACs can be caged with photoremovable groups.^23^^,^^24^ These systems allow efficient photocleavage upon light irradiation, showcasing their potential for precise regulation.^25^ Additionally, the photoisomerization strategy has been employed to control the *trans-*to*-cis* transformation of BRD4 PROTACs. Under 415 nm light irradiation, *trans*-photo PROTAC was activated to degrade BRD2 efficiently, while reverse activation was achieved with 530 nm light irradiation. This dual regulation was significantly influenced by the distance between the two warheads, highlighting the importance of spatial dynamics in PROTAC activity.^26^ These optochemically controlled PROTACs represent a promising approach for real-time regulation of protein degradation activity. Yet, the CDK4/6-targeted opto-PROTAC remains to be developed to realize spatiotemporal control of cell-cycle arrest.

**Figure 1:**
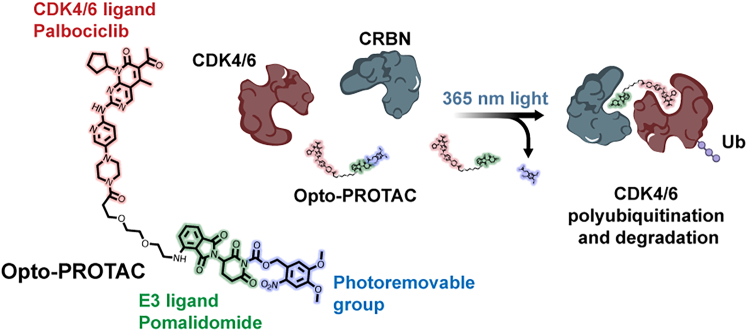
Schematic illustration of Opto-PROTAC The CRBN E3 ligand was caged with a photoremovable group, which can block its interaction with CRBN. Light irradiation can cleave the photoremovable group and activate Opto-PROTAC, which hijacks the target proteins and induces CDK4/6 polyubiquitination and degradation. The figure was created with BioRender.com.

Herein, we developed an optochemically controlled PROTAC targeting CDK4/6 (Opto-PROTAC), by caging a CDK4/6 PROTAC with a 4, 5-dimethoxy-2-nitrobenzyl (DMNB) group at the glutarimide NH of pomalidomide, which can significantly block its interaction with CRBN ligase.^27^ Upon 365 nm light irradiation, Opto-PROTAC will be cleaved and release free CDK4/6 PROTAC, triggering CDK4/6-targeted degradation to induce G1 cell-cycle arrest (Figure 1). This light-controlled system allows spatiotemporal CDK4/6 degradation and G1 cell-cycle arrest, enabling a promising strategy to enhance specificity for cancer treatment and fundamental biological research.

## Results

### CDK4/6 Opto-PROTAC exhibits photolysis capability

Based on the structure of DDB1-CRBN ubiquitin ligase in complex with pomalidomide (PDB ID: 4CI3), we found the glutarimide NH group of the pomalidomide moiety played a very essential role in interacting with His380 of the CRBN protein and forming a hydrogen bond between these two molecules (Figure S1). Therefore, we hypothesized that the incorporation of a bulky DMNB group to the glutarimide nitrogen can block the interaction between PROTAC and CRBN. Hence, we synthesized a CDK4/6 PROTAC based on our previous protocol and conjugated DMNB photoremovable molecule on the glutarimide nitrogen (Figure S2).^4^ The structures of the final product Opto-PROTAC and PROTAC as a control group were validated by H^1^-NMR and LC-MS (Figures S3–S6).

**Figure 2:**
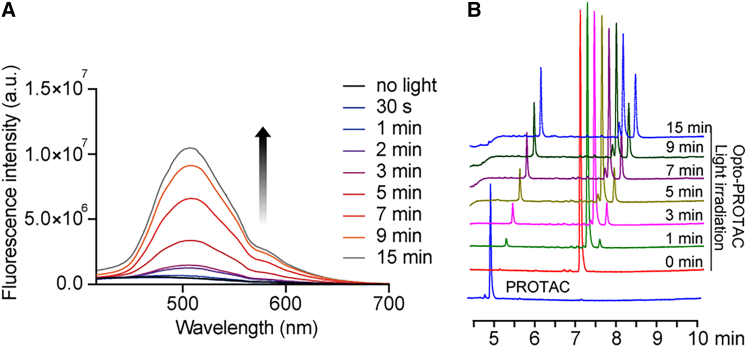
Optochemical properties of Opto-PROTAC (A) Fluorescence spectra of Opto-PROTAC after light irradiation. The indicated drugs were dissolved in H_2_O at a concentration of 100 μM and 365 nm light (10 mW/cm^2^) was applied. The excitation wavelength was 400 nm, with emission wavelength from 416 nm to 700 nm. (B) HPLC analysis of Opto-PROTAC, showing a clear photochemical conversion to desired product. The drugs were dissolved in aqueous solutions of acetonitrile and water (1:1, v/v) at a concentration of 100 μM. Light irradiation (365 nm, 10 mW/cm^2^) was applied for different irradiation time periods.

Next, we investigated the photochemical behavior using UV-vis spectroscopy. Light at 365 nm (10 mW/cm^2^) was applied to irradiate the molecules in aqueous solutions for different time periods. And a series of fluorescence spectra were characterized with excitation wavelength at 400 nm and emission wavelength in the range of 416–700 nm. Surprisingly, a significantly increasing fluorescence intensity at 510 nm was observed after light irradiation, indicating the light responsiveness of the Opto-PROTAC prodrug (Figure 2A). The photolysis reaction of the prodrug upon light irradiation was further verified by the high-performance liquid chromatography (HPLC) analysis (Figure 2B). The HPLC result clearly showcased the appearance of free PROTAC and the decrease of the Opto-PROTAC peak with prolonged light irradiation, indicating that Opto-PROTAC can be cleaved by light to generate free PROTAC in a time-dependent manner. In addition, the stability of Opto-PROTAC in the dark was also examined in the PBS for 48 h. Approximately 80% of the prodrug remained intact, and PROTAC release was not detectable (Figure S8).

### Opto-PROTAC mediates CDK4/6 degradation upon light irradiation

**Figure 3:**
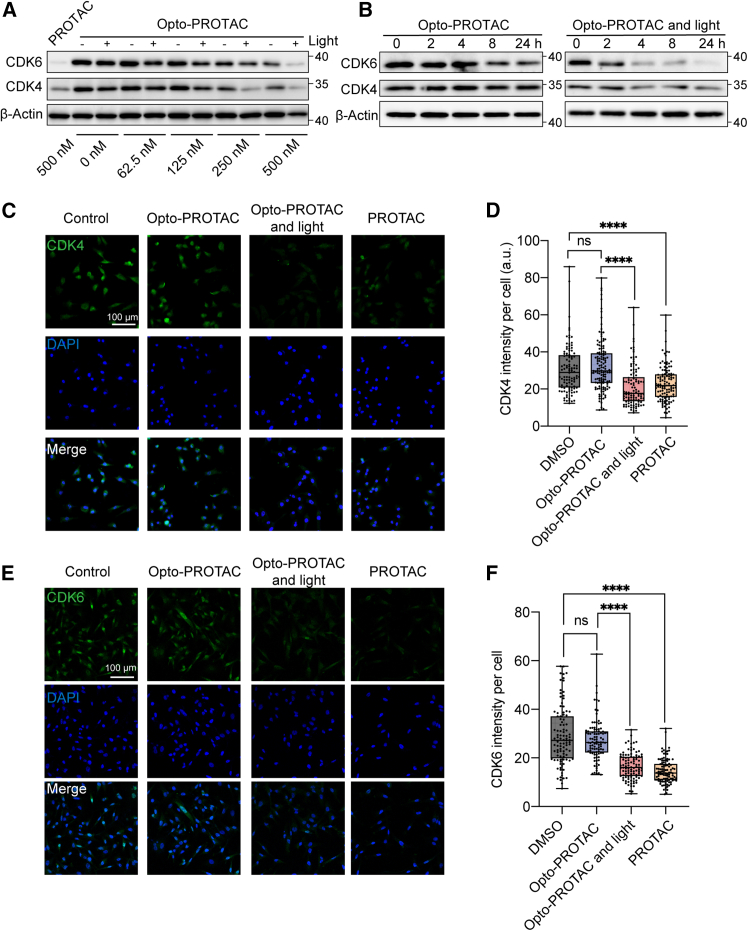
Light-triggered CDK4/6 degradation by Opto-PROTAC (A) Representative western blot results showing CDK4/6 protein levels in MDA-MB-231 cells at 24 h after treatment with different drug doses, with or without light irradiation (365 nm, 10 mW/cm^2^, 5 min) (*n* = 3). (B) Western blot analysis of CDK4/6 protein levels in MDA-MB-231 cells treated with Opto-PROTAC (500 nM) with or without light irradiation after different time periods (*n* = 3). (C–F) Immunofluorescence analysis of CDK4 and CDK6 proteins at 24 h after drug with or without light treatment (365 nm, 10 mW/cm^2^, 5 min) and calculated fluorescence intensity per cell. Scale bars: 100 μm. Data in (D) and (F) represent median, first, and third quartiles, minimum and maximum of at least 100 cells per group. ∗∗∗∗*p* < 0.0001. *p* value is calculated using one-way ANOVA with Tukey’s multiple comparison test.

After demonstrating a photochemical behaviour of Opto-PROTAC, we further investigated its functionality in a biological context. MDA-MB-231 triple negative breast cancer cells were chosen due to their high sensitivity to CDK4/6 inhibition.^28^ The cells were treated with DMSO (control group), PROTAC, and Opto-PROTAC, followed by 365 nm light irradiation (10 mW/cm^2^) for irradiation groups after 1 h. The cells were harvested, and proteins were collected after 24 h for western blot analysis. PROTAC (500 nM) treatment significantly decreased the CDK4 and CDK6 protein levels compared with the control group, implying that this PROTAC can mediate the degradation of both CDK4 and CDK6 proteins (Figure 3A). Interestingly, for the cells incubated with Opto-PROTAC, protein degradation occurred in a light-dependent manner. Upon light irradiation, Opto-PROTAC induced pronounced reduction of the CDK4/6 level, compared with the non-irradiation groups. We also identified that for the cells treated with Opto-PROTAC, light irradiation can decrease the CDK4/6 level in a time-dependent manner. Starting from 4 h after the treatment, activated Opto-PROTAC can induce CDK4/6 degradation, while the protein levels remained almost unchanged in the non-irradiation group (Figure 3B). Immunofluorescence analysis was performed to further verify the light-responsive protein degradation effect. Qupath software was utilized for calculating the fluorescence intensity per cell.^29^ Similar to the PROTAC treatment, Opto-PROTAC with light irradiation significantly decreased CDK4 and CDK6 fluorescence level in the cells. In contrast, the fluorescence intensity did not notably change when the cells were treated with Opto-PROTAC in the dark (Figures 3C–3F). These results demonstrated that CDK4/6 degradation can be controlled by light using this Opto-PROTAC. Upon light irradiation, this prodrug can be cleaved and activated to induce a dramatic decrease of both CDK4 and CDK6 proteins.

### Opto-PROTAC mediates light-triggered G1 cell-cycle arrest

**Figure 4:**
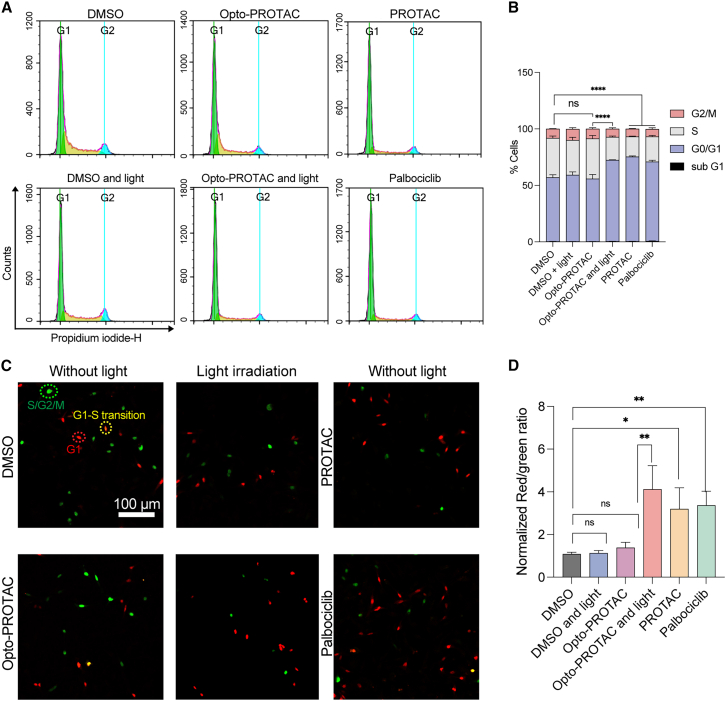
Optochemically controlled G1 cell-cycle arrest by Opto-PROTAC (A and B) Cell-cycle analysis of the cells treated with indicated drugs (2 μM) with or without light irradiation (365 nm, 10 mW/cm^2^, 5 min). At 24 h after the treatment, the cells were fixed and analyzed by flow cytometry (*n* = 3). The results were quantified in (B). (C and D) The FUCCI reporter system analysis for the cells treated with different drugs with or without light irradiation (365 nm, 10 mW/cm^2^, 5 min) and statistical analysis. Scale bars: 100 μm. Data in (B) and (D) represent mean ± SD. of three independent experiments. ∗*p* < 0.05, ∗∗*p* < 0.01, and ∗∗∗∗*p* < 0.0001. *p* value is calculated using one-way ANOVA with Tukey’s multiple comparison test.

During cell-cycle progression, CDK4/6 proteins form protein complexes together with cyclin D, which subsequently phosphorylate Rb protein, thereby inactivating this protein and releasing it from transcription factor E2F. Hence, E2F is activated to initiate the transcription of different cell cycle-regulating genes to promote cell-cycle progression. Therefore, we investigated whether this light-induced CDK4/6 degradation system can be utilized for light-triggered cell-cycle arrest. MDA-MB-231 cells were treated with 2 μM PROTAC, Opto-PROTAC, or palbociclib, followed by light irradiation for irradiation groups. After 24 h incubation, the cells were fixed and stained with propidium iodide (PI) cell-cycle indicator and analyzed by flow cytometry. The percentage of cells in the G1 phase increased to 70% following exposure to Opto-PROTAC and light irradiation, comparable to the cells treated with free PROTAC and palbociclib. As a control, the cells treated with Opto-PROTAC were maintained in the dark for the same time period. We found that about 56% of cells were in the G1 phase, which was not statistically different from cells treated with DMSO only, indicating that the uncaging of Opto-PROTAC is negligible to induce cell-cycle change in the absence of light (Figures 4A and 4B). To exclude the influence of light treatment we used for photocleavage on cell-cycle regulation, we exposed the DMSO-treated cells to the same light irradiation. As shown in the figure, the cells exposed to light did not exhibit notable difference compared with the control group, implying that the cell-cycle regulation was not disturbed by light treatment (Figures 4A and 4B).

### Spatiotemporal regulation of G1 cell-cycle arrest can be achieved by Opto-PROTAC

To further validate the ability of this Opto-PROTAC system to spatiotemporally control cell cycle, we stably expressed fluorescent ubiquitination-based cell-cycle indicator (FUCCI) in MDA-MB-231 cells. The monomeric Kusabira Orange 2 (mKO2)-hCDT1 and monomeric Azami Green (mAG)-hGEM reporter system was chosen to visualize G1 cells with red fluorescence signal in the nuclei (Figure 4C).^30^ Hence, the cell-cycle progression of each cell can be visualized and tracked through FUCCI reporter system, allowing cell-cycle investigation at the single cell level and in a timely manner. The cells were treated with indicated drugs and exposed to 365 nm light after 1 h or maintained in the dark. The cells were visualized after 24 h and the number of the cells with red or green nucleus was calculated. And the red-to-green ratio was calculated and normalized to the DMSO group. As expected, the cells treated with Opto-PROTAC and exposed to light irradiation exhibited a higher ratio of red fluorescent signal. In contrast, Opto-PROTAC treatment did not notably influence the red-to-green ratio for the cells maintained in the dark compared with the control group, implying that the caging group was effective in blocking the bioactivity of this PROTAC to regulate cell cycle (Figures 4C and 4D).

**Figure 5:**
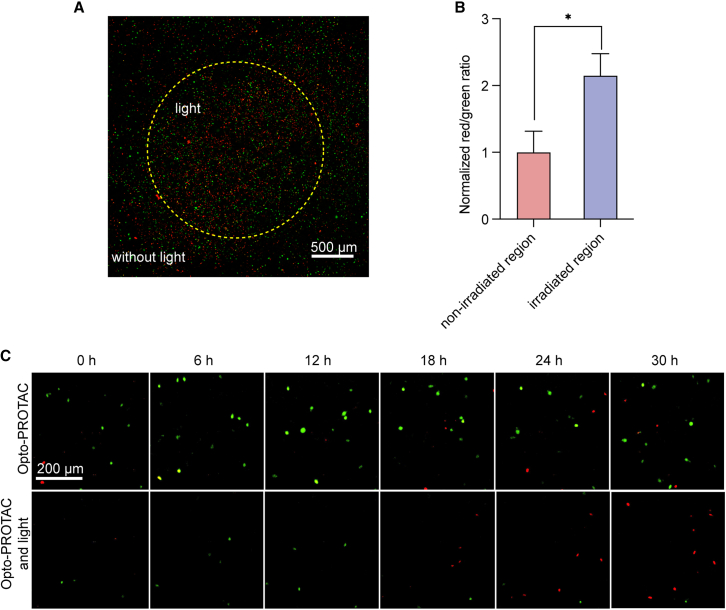
Spatiotemporal control of G1 cell-cycle arrest by Opto-PROTAC (A and B) The spatiotemporal control of cell cycle by Opto-PROTAC. The FUCCI stably expressed MDA-MB-231 cells were treated with Opto-PROTAC (2 μM), and light irradiation (365 nm, 10 mW/cm^2^, 5 min) was applied in the center of the cell culture dish. At 24 h after incubation, the cells were analyzed by CLSM. The result was also quantified in (B). Scale bars: 500 μm. (C) The FUCCI reporter system analysis of cells treated with indicated drugs with or without light irradiation after different time periods using Cytosmart device. Scale bars: 200 μm. Data in (B) represent mean ± SD. of three independent experiments. ∗*p* < 0.05. *p* value is calculated using one-way ANOVA with Tukey’s multiple comparison test.

To further validate the precise cell-cycle control by light irradiation, the cells transduced with FUCCI reporter system were treated with Opto-PROTAC, and light was only applied to the center of the cell culture dish after 1 h. At 24 h after incubation, the cells were visualized with confocal laser scanning microscopy (CLSM) and normalized red-to-green ratio was calculated. Interestingly, we found that the percentage of cells with red nucleus was notably higher in the irradiated region (indicated by the yellow circle), compared with the non-irradiated region (Figures 5A and 5B). This result indicated that although the cells in different regions were exposed to Opto-PROTAC, cell-cycle arrest was highly induced in the irradiated cells where photocleavage occurred. After treatment with Opto-PROTAC, we also traced the real-time fluorescence change of the cells exposed to light irradiation or kept in the dark. The percentage of red-fluorescent nuclei increased dramatically in a time-dependent manner in the cells treated with both Opto-PROTAC and light irradiation, while no increase was observed in the non-irradiated cells, indicating that light irradiation can be applied timely to trigger cell-cycle arrest (Figure 5C). In summary, these results showcased that this Opto-PROTAC for CDK4/6 can be utilized to regulate G1 cell-cycle arrest timely and precisely at desired location.

## Discussion

CDK4/6-mediated Rb phosphorylation and G1/S phase transition play critical roles in cancer cell proliferation. Therefore, the dysregulated CDK4/6-Rb axis remains a high-value therapeutic target in oncology. Yet, conventional CDK4/6 inhibition and degradation for cell-cycle arrest is strongly restricted in clinical applications due to suboptimal therapeutic efficacy and unwanted adverse effects. In addition, both conventional functions of CDK6 as kinase and transcriptional regulator require nuclear localization. Recent studies using fluorescence imaging indicate that CDK6 is primarily localized in the cytoplasmic edges and extensions of cells, regulating actin dynamics and cell motility.^16^ Yet, detailed investigation of location-function relationship of CDK4/6 remains elusive, which requires precise and timely control of CDK4/6 at sub-cellular level. Therefore, it is highly desired to develop research tools to spatiotemporally control CDK4/6 activity to enhance therapeutic specificity and identify non-classical functions of CDK4/6 that could be related to the cellular distribution.

Prior optical approaches for cell-cycle regulation rely on unspecific or complicated processes. A photocaged proteasome inhibitor MG-132 was developed to widely block proteasomal degradation of multiple cell-cycle regulators, enabling optical control of cell-cycle arrest at metaphase.^21^ Optogenetic tools were also constructed to regulate p21 protein activation or spindle assembly checkpoint, enabling spatiotemporal cell-cycle control, which, however, requires complicated protein expression system that might hinder clinical implementation.^31^^,^^32^ In this study, we employed PROTACs for CDK4/6 degradation and developed an Opto-PROTAC molecule by masking the CRBN ligand with DMNB photoremovable protecting group. The fluorescence spectra and HPLC analysis indicated efficient photocleavage of Opto-PROTAC and release of active PROTAC upon light irradiation. To further illustrate the spatiotemporal control of G1 cell-cycle arrest, the FUCCI reporter system was employed to show G1/S phase transition at the single-cell level. By coupling with localized light irradiation and time-relapse Cytosmart imaging system, we successfully demonstrated the ability of Opto-PROTAC for spatiotemporal control of CDK4/6 degradation and G1 cell-cycle arrest.

In conclusion, we developed an Opto-PROTAC molecule to specifically induce light-triggered CDK4/6 protein degradation. Upon light irradiation, Opto-PROTAC can be cleaved to release free PROTAC, inducing the ubiquitination and proteasomal degradation of CDK4/6 and G1 cell-cycle arrest for enhanced specificity in cancer therapy. We demonstrated that Opto-PROTAC can also spatiotemporally block cell-cycle progression, which can provide a promising research tool for fundamental biological investigations in cell-cycle dynamics.

### Limitations of the study

While our study provides proof of concept for spatiotemporal CDK4/6 degradation using the Opto-PROTAC system, several limitations warrant discussion. First, the current validation is limited to *in vitro* models under controlled light exposure. Translating this approach to *in vivo* settings requires addressing challenges such as tissue penetration of 365 nm UV light and systemic pharmacokinetics of Opto-PROTAC molecules. Long-wavelength photoremovable groups or upconversion nanoparticle systems could facilitate further translation. In addition, the study focuses on canonical cell-cycle arrest function of CDK4/6 but not non-classical roles such as cytoskeleton regulation. Exploiting Opto-PROTAC to spatiotemporally control CDK4/6 degradation at the sub-cellular level may help understand location-function relationship of non-classical functions of CDK4/6.

## Resource availability

### Lead contact

Further information and any requests should be directed to and will be fulfilled by the lead contact, Prof. Weiping Wang (wangwp@hku.hk).

### Materials availability

Chemical reagents used in this study can be shared upon reasonable request to the lead contact.

### Data and code availability

• All data reported in this paper will be shared by the lead contact upon request.
• This paper does not report original code.
• Any additional information required to reanalyze the data reported in this paper is available from the lead contact upon request.

## Acknowledgments

We acknowledge the assistance of the Faculty Core Facility of Li Ka Shing Faculty of Medicine, The University of Hong Kong.

This work was supported by the 10.13039/501100001809National Natural Science Foundation of China (Excellent Young Scientists Fund, no. 82222903), 10.13039/501100002858China Postdoctoral Science Foundation (no. 2024M763792), Research Foundation of Medical Science and Technology of Guangdong Province (no. A2025003), and Fostering Program for NSFC Young Applicants (Tulip Talent Training Program) of 10.13039/501100018573Sun Yat-sen University Cancer Center (no. 2025yfd05).

## Author contributions

W.W., L.L., and T.W. conceived the concept of this study. T.W., Y.Z., and Y.L. performed the experiments and data acquisition. T.W., Y.Z., L.W., L.L., and W.W. contributed to the interpretation of the results and the writing of this manuscript. All authors have given approval to the final version of the manuscript.

## Declaration of interests

The authors declare no competing interests.

## STAR★Methods

### Key resources table

**Table:** 

REAGENT or RESOURCE	SOURCE	IDENTIFIER
**Antibodies**		

CDK4	Cell signaling technology	Cat# 12790; RRID: AB_2631166
CDK6	Cell signaling technology	Cat# 13331; RRID: AB_2721897
β-Actin	Cell signaling technology	Cat# 4967; RRID: AB_330288
Goat Anti Mouse (IgG) secondary antibody HRP	Abcam	Cat# ab6789; RRID: AB_955439
Goat Anti Rabbit (IgG) secondary antibody HRP	Abcam	Cat# ab6721; RRID: AB_955447
Goat Anti Rabbit (IgG) secondary antibody FITC	Abcam	Cat# ab6717; RRID: AB_955238

**Chemicals, peptides, and recombinant proteins**		

*tert*-Butyl 3-[2-(2-(2-aminoethoxy)ethoxy)ethoxy]propionate	MCE	Cat# HY-135804
N-ethyl-N-propan-2-ylpropan-2-amine	TCI	Cat# D1599
2-(2,6-dioxopiperidin-3-yl)-4-fluoroisoindoline-1,3-dione	MCE	Cat# HY-41547
Sodium hydride	Sigma	Cat# 452912
(4,5-dimethoxy-2-nitrophenyl)methyl carbonochloridate	Sigma	Cat# 420069
2,2,2-Trifluoroacetic acid	Sigma	Cat# 808260
[dimethylamino-(3-oxidotriazolo[4,5-*b*]pyridin-3-ium-1-yl)methylidene]-dimethylazanium; hexafluorophosphate	MCE	Cat# HY-Y1703
Palbociclib	MCE	Cat# HY-50767
MTT	MCE	Cat# HY-15924
Puromycin	GoldBio	Cat# P-600-100
RIPA buffer	Thermo Scientific	Cat# 89900
Clarity Western ECL Substrate	BioRad	Cat# 1705061

**Critical commercial assays**		

Pierce BCA protein assay kit	Thermo Scientific	Cat# 23225
FxCycle PI/RNase staining solution	Thermo Scientific	Cat# F10797

**Experimental models: Cell lines**		

MDA-MB-231	ATCC	HTB-26
HEK-293T	ATCC	CRL-3216

**Recombinant DNA**		

pBOB-EF1-FastFUCCI-Puro	Addgene	RRID: Addgene_86849
psPAX2	Addgene	RRID: Addgene_12260
pMD2.G	Addgene	RRID: Addgene_12259

**Software and algorithms**		

Graphpad prim 8		http://www.Graphpad.com
Pymol		https://www.pymol.org/

### Experimental model and study participant details

#### Cell lines and cultures

The human triple negative breast cancer cell line (MDA-MB-231) and human embryonic kidney 293 cell line with SV40 large T antigen (HEK-293T) were used in this study. High glucose (4.5 g/mL) Dulbecco modified Eagle medium (DMEM, Gibco) containing 10% fetal bovine serum (FBS, Gibco) and 1% penicillin/streptomycin (Gibco) were used for cell culture. The cells were incubated in the incubator under 100% humidity, 5% CO_2_, 37 °C condition.

### Method details

#### Design and synthesis of the Opto-PROTAC

To synthesize compound 1, the *tert*-Butyl 3-[2-(2-(2-aminoethoxy)ethoxy)ethoxy]propionate (86.19 mg, 1.0 equivalent) was dissolved in dry DMSO (4 mL) and DIPEA (126 μL, 2.0 equivalent) and the CRBN ligand, 2-(2,6-dioxopiperidin-3-yl)-4-fluoroisoindoline-1,3-dione (102 mg, 1.0 equivalent) was added. The mixture was stirred at 90 °C for 18 h. After cooling, it was poured onto saturated brine (100 mL) and extracted with EtOAc (2 × 50 mL). The combined organic layers were washed with brine (50 mL), dried over Na_2_SO_4_, filtered and concentrated.

To a solution of compound 1 (22.89 mg, 1.0 equivalent) in DMF (1 mL) was added NaH (3.74 mg, 60% in mineral oil, 2.0 equivalent) at 0°C. After stirring for 10 min, (4,5-dimethoxy-2-nitrophenyl)methyl carbonochloridate (25.94 mg, 2.0 equivalent) was added to the mixture at 0°C. The reaction mixture was then warmed up to room temperature and stirred for additional 3 hours. The resulting mixture was purified by preparative HPLC (10 to 100% acetonitrile/0.1% TFA in H_2_O) to afford desired product, compound 2.

To a solution of compound 2 (11.0 mg, 1.0 equivalent) in CH_2_Cl_2_ (2 ml) was added TFA (1 mL). After stirring for 2 h at 40 °C, the resulting mixture was concentrated under reduced pressure. The product (15.3 mg, 1.0 equivalent) was dissolved in DMF (1 mL) followed by addition of DIPEA (16.2 μL, 4.0 equivalent), HATU (9.72 mg, 1.1 equivalent) and Palbociclib (10.39 mg, 1.0 equivalent). After stirring at room temperature for 24 h, the resulting mixture was poured onto saturated brine (100 mL) and extracted with EtOAc (2 × 50 mL). The combined organic layers were washed with brine (50 mL), dried over Na_2_SO_4_, filtered and concentrated. The resulting mixture was purified by preparative HPLC (10 to 100% acetonitrile/0.1% TFA in H_2_O) to afford desired product.

#### Characterization of the photolysis reaction

The change of fluorescence spectrum of 100 μM Opto-PROTAC after light irradiation (365 nm, 10 mW/cm^2^) was characterized by UV-Vis spectrophotometer. The excitation wavelength was 400 nm for the fluorescence spectrum. And the emission wavelength ranged from 416 nm to 700 nm. The photolysis reaction was also characterized by HPLC analysis. Opto-PROTAC was dissolved in aqueous solution of acetonitrile and water (1:1, v/v) at the final concentration of 100 μM, and irradiated with light for 1 min, 3 min, 5 min, 7 min, 9 min, and 15 min to measure the cleavage of Opto-PROTAC and the release of PROTAC.

#### Western blot

The MDA-MB-231 cells were seeded in 12-well plates at 1 × 10^5^ density and incubated for 24 h. Then the cells were treated with indicated drugs, followed by light irradiation (365 nm, 10 mW/cm^2^, 5 min) 1 h after drug incubation for irradiation groups. 24 h after the drug treatment, the cells were washed with 1x PBS three times and harvested with RIPA buffer (supplemented with Halt protease and phosphatase inhibitor cocktail, Thermo Scientific). Total protein concentration was determined by pierce BCA protein assay kit (Thermo scientific, #23225). Equal amounts of proteins were separated by the SDS-PAGE electrophoresis and transferred to a nitrocellulose membrane through the wet transfer system. The membranes were blocked with 5% BSA in TBST for 1 h at room temperature and incubated with primary antibodies at 4 °C overnight. After washed with TBST three times, the membranes were incubated with secondary antibodies for 1 h at room temperature. After washed with TBST three times, the antibody staining was visualized by Clarity Western ECL Substrate (BioRad) and captured by ChemiDoc Imaging System (BioRad).

#### Cell cycle analysis by flow cytometry

The cells were seeded in 12-well plates at 1 × 10^5^ density and incubated for 24 h, followed by drug treatment and light irradiation. 24 h after treatment, the cells were washed with 1xPBS and harvested. The cells were fixed with cold 75% ethanol overnight. After washing with PBS, the cells were stained with FxCycle™ PI/RNase staining solution (Thermo Fisher, F10797) and analyzed by Agilent NovoCyte Quanteon analyzer.

#### Immunofluorescence analysis

The cells were seeded on Lab-Tek Chamber Slide (Thermo scientific) for 24 h, followed by treatment of indicated drugs and light irradiation. After 24 h incubation, the cells were washed with PBS three times and fixed in 3.7% paraformaldehyde solution in PBS for 10 min. After washed with PBS three times, the cells were stained with DAPI solution (Thermo scientific, D1306) and mounted with SlowFade Diamond Antifade Mountant (Thermo scientific, S36963). The Zeiss LSM 900 inverted confocal microscope equipped with 10 × 0.45 NA, 20 × 0.8 NA and 40 × 1.4 NA lens was used for detecting and capturing fluorescence signal. The same exposure settings were used across all conditions in each individual experiment. The fluorescence intensity in each cell was detected and measured by QuPath software.

#### Plasmid transduction

The plasmid pBOB-EF1-FastFUCCI-Puro was a gift from Kevin Brindle & Duncan Jodrell (Addgene plasmid # 86849; http://n2t.net/addgene:86849; RRID:Addgene_86849). psPAX2 was a gift from Didier Trono (Addgene plasmid # 12260; http://n2t.net/addgene:12260; RRID: Addgene_12260). pMD2.G was a gift from Didier Trono (Addgene plasmid # 12259; http://n2t.net/addgene:12259; RRID: Addgene_12259). For stable transfection, the lentivirus reagents were produced by transduction in 293T cells with indicated plasmids and helper plasmids psPAX2 and pMD2.G. Sixteen hours after transduction, the cell culture medium was changed to complete DMEM medium. Supernatant was collected 48 hours and 72 hours after transduction, filtered with a 0.45 μm low-bind filter, and frozen at -80°C until use. For lentiviral infection, MDA-MB-231 cells were plated at 70% density and treated with the lentivirus supernatant. The positive cells were selected using 1 μg/mL puromycin (GoldBio) for 2 weeks.

### Quantification and statistical analysis

GraphPad Prism 8.0 software (GraphPad Software, Inc) was used for statistical data analysis. To analyze and compare the differences among multiple-group means, one-way ANOVA was used with Tukey’s multiple comparison test. To compare the differences between two groups, two-tailed unpaired Student’s t-test was used. Values of *p* < 0.05 were considered significant. Results are represented as means ± SD.
