# Supplementary material for: Optochemical control of G1 cell cycle by regulating CDK4/6 degradation
Source: iScience. 2025 Aug 6;28(9):113304. doi: 10.1016/j.isci.2025.113304 (PMC12398220; doi:10.1016/j.isci.2025.113304)
Supplement: Document S1. Figures S1–S8 [file mmc1.pdf]

iScience, Volume *28*

Supplemental information

Optochemical control of G1 cell cycle by regulating CDK4/6 degradation

Tianyi Wang, Yaming Zhang, Yuwei Liu, Lichao Wang, Lijun Liu, and Weiping Wang

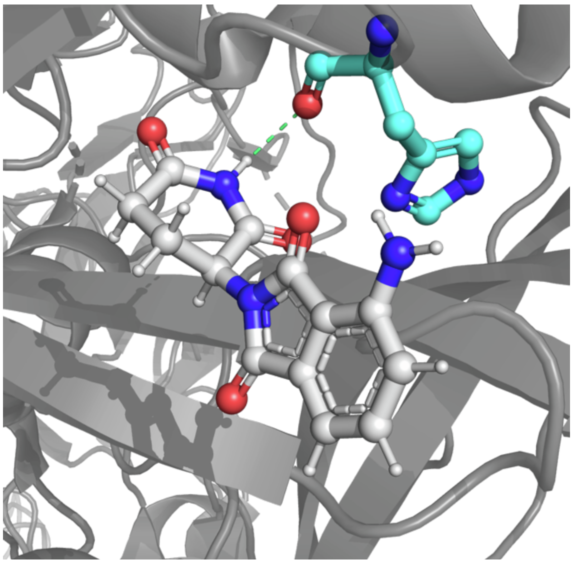

Figure S1. The structure of the DDB1-CRBN E3 ubiquitin ligase in complex with pomalidomide (Protein Data Bank: 4CI3).

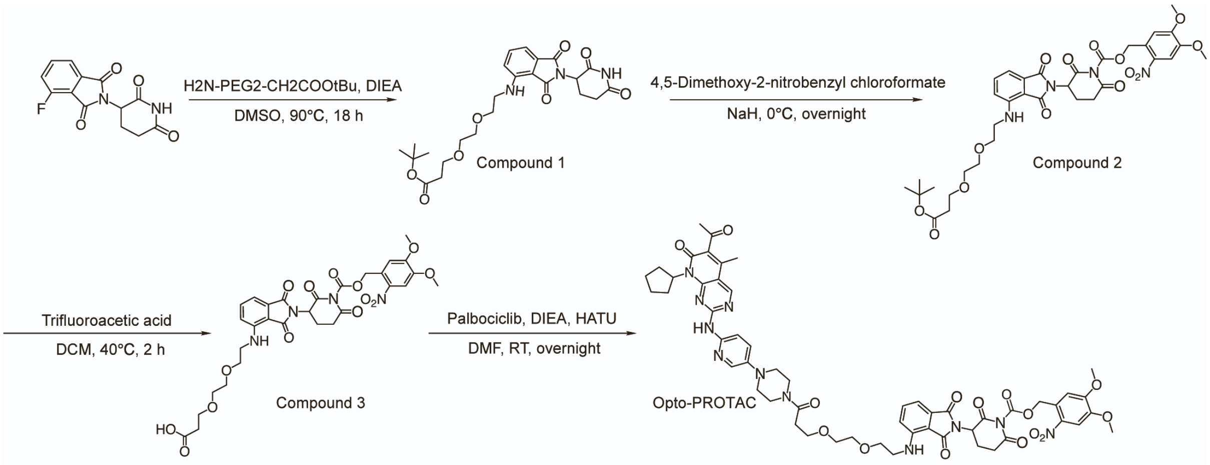

Figure S2. The synthetic route of Opto-PROTAC.

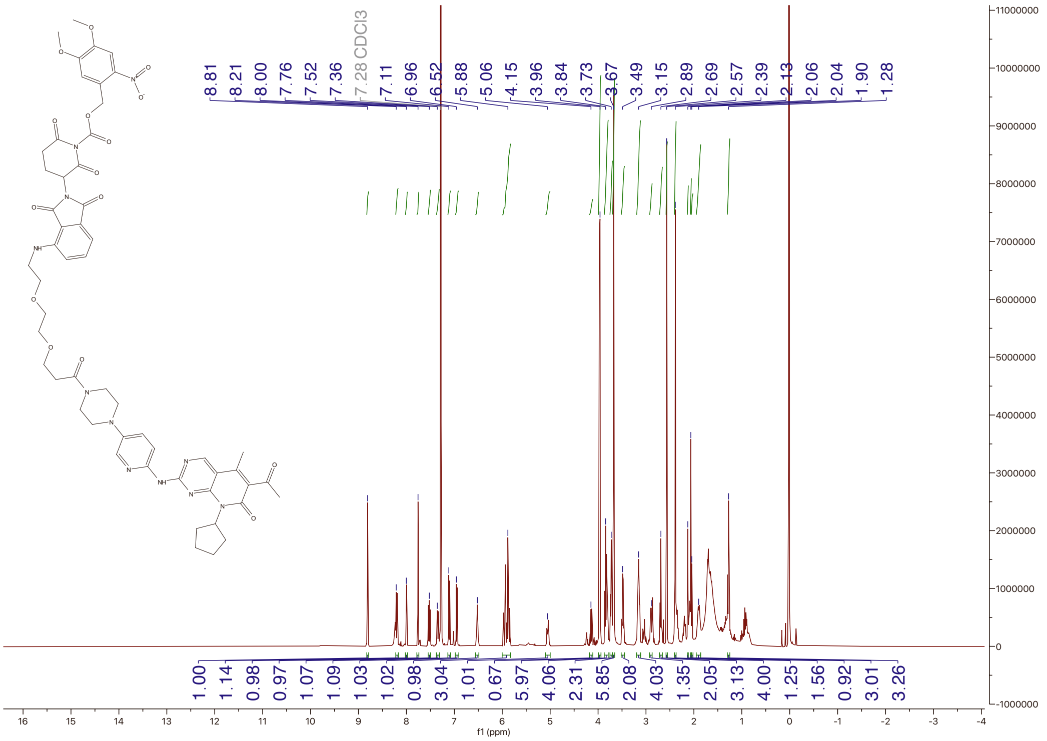

Figure S3. ^{1}H-NMR spectrum of Opto-PROTAC. ^{1}H-NMR (500 MHz, $CDCl_3$) δ (ppm): 8.81 (s, 1H), 8.20 (d, *J* = 9.1 Hz, 1H), 8.00 (d, *J* = 2.9 Hz, 1H), 7.76 (s, 1H), 7.55 – 7.50 (m, 1H), 7.34 (dd, *J* = 9.1, 3.0 Hz, 1H), 7.11 (d, *J* = 7.1 Hz, 1H), 6.95 (d, *J* = 8.6 Hz, 1H), 6.52 (t, *J* = 5.7 Hz, 1H), 6.00 – 5.83 (m, 3H), 5.05 (dd, *J* = 12.0, 5.5 Hz, 1H), 4.14 (q, *J* = 7.1 Hz, 1H), 3.97 (d, *J* = 5.6 Hz, 6H), 3.83 (dt, *J* = 11.0, 5.9 Hz, 4H), 3.73 (t, *J* = 5.4 Hz, 2H), 3.67 (s, 6H), 3.48 (q, *J* = 5.5 Hz, 2H), 3.15 (dt, *J* = 10.3, 5.1 Hz, 4H), 2.90 (q, *J* = 3.3 Hz, 1H), 2.69 (t, *J* = 6.5 Hz, 2H), 2.57 (s, 3H), 2.39 (s, 4H), 2.13 (s, 1H), 2.06 (s, 2H), 2.04 (s, 1H), 1.95 – 1.86 (m, 3H), 1.30 – 1.25 (m, 3H).

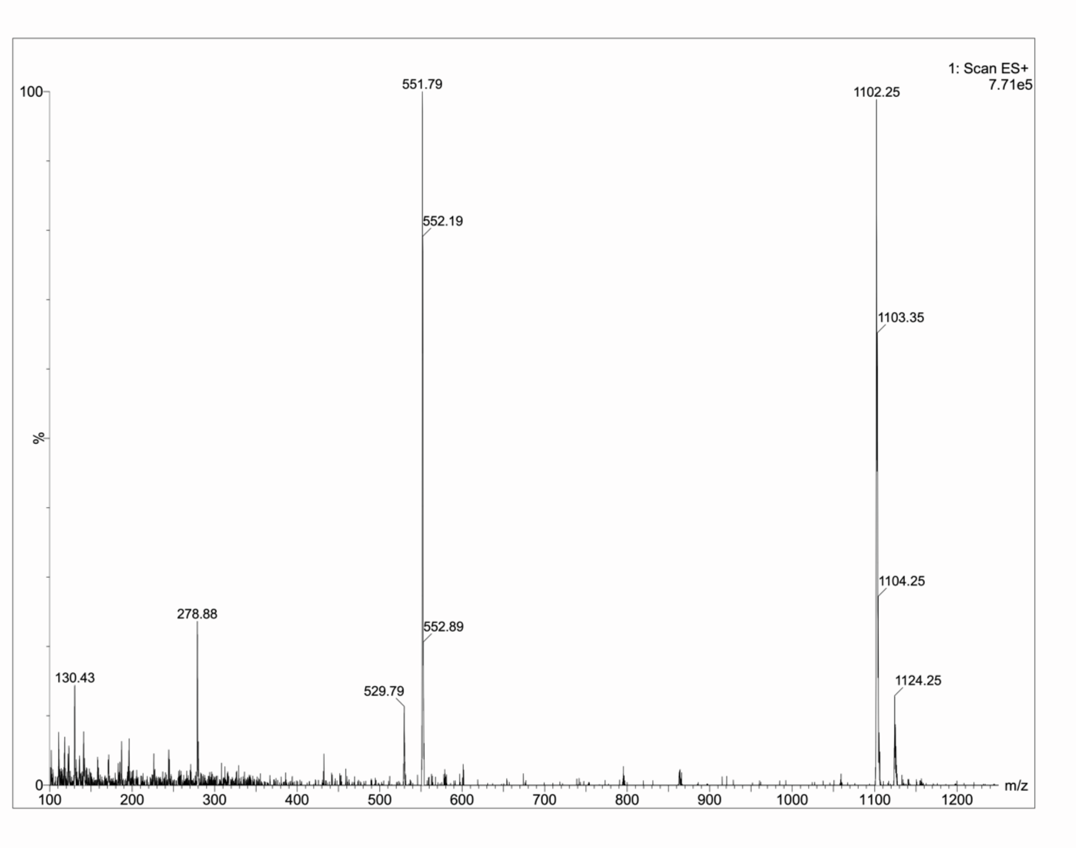

Figure S4. Mass spectrometry of Opto-PROTAC ($C_{54}H_{59}N_{11}O_{15}$). $[M + H]^+$ calculated: 1103.13; observed: 1102.25.

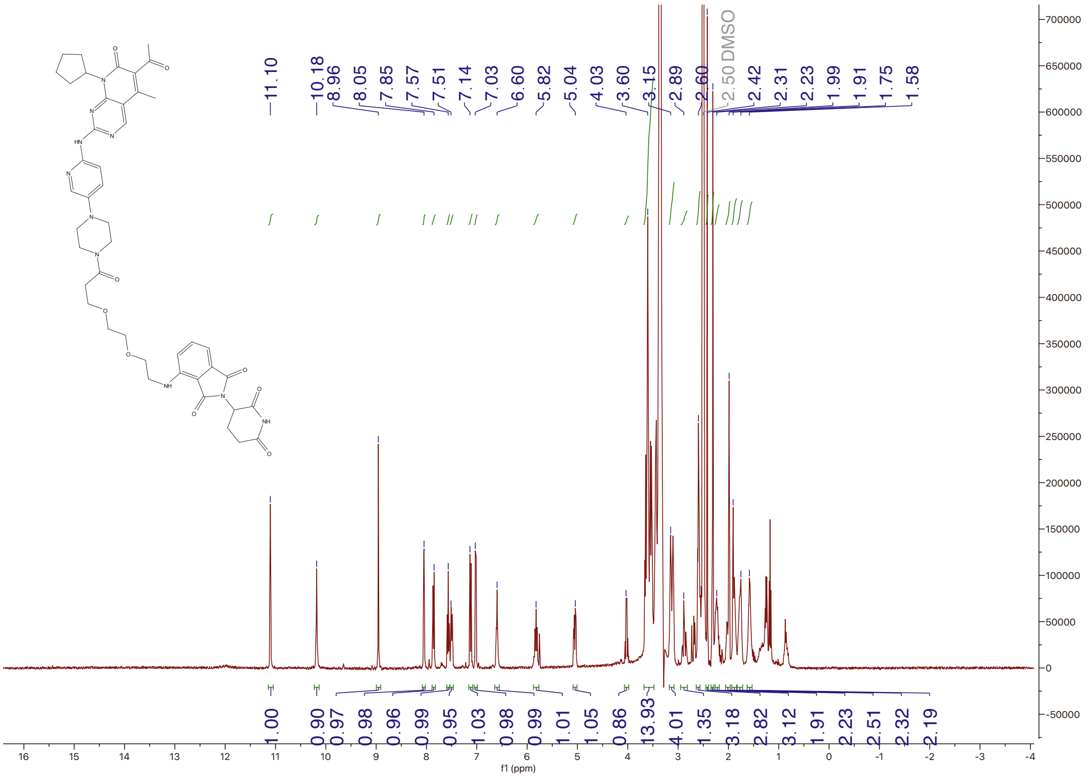

Figure S5. ^{1}H-NMR spectrum of PROTAC. ^{1}H-NMR (500 MHz, DMSO-d6) δ (ppm): 11.10 (s, 1H), 10.18 (s, 1H), 8.96 (s, 1H), 8.05 (d, J = 2.8 Hz, 1H), 7.86 (d, J = 9.0 Hz, 1H), 7.57 (t, J = 7.8 Hz, 1H), 7.50 (dd, J = 9.0, 3.0 Hz, 1H), 7.12 (d, J = 8.6 Hz, 1H), 7.02 (d, J = 7.0 Hz, 1H), 6.60 (t, J = 5.9 Hz, 1H), 5.87 – 5.73 (m, 1H), 5.05 (dd, J = 13.0, 5.4 Hz, 1H), 4.03 (q, J = 7.1 Hz, 1H), 3.68 – 3.49 (m, 14H), 3.17 – 3.06 (m, 4H), 2.88 (td, J = 15.4, 5.4 Hz, 1H), 2.59 (q, J = 6.9 Hz, 3H), 2.42 (s, 3H), 2.31 (s, 3H), 2.24 (s, 2H), 2.03 (s, 1H), 1.99 (s, 2H), 1.91 (s, 2H), 1.77 (d, J = 11.4 Hz, 2H), 1.58 (d, J = 5.5 Hz, 2H).

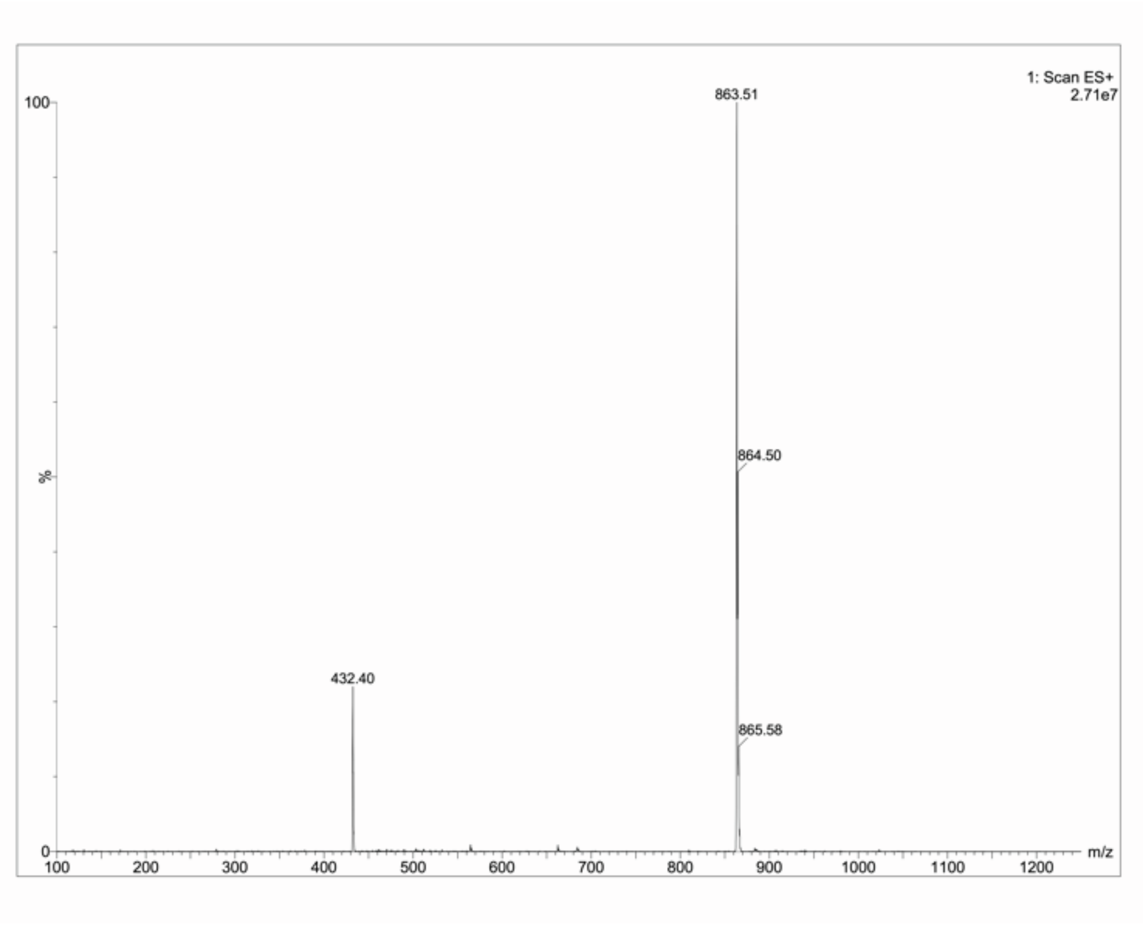

Figure S6. Mass spectrometry of PROTAC ($C_{44}H_{50}N_{10}O_9$). $[M + H]^+$ calculated: 863.95; observed: 863.51.

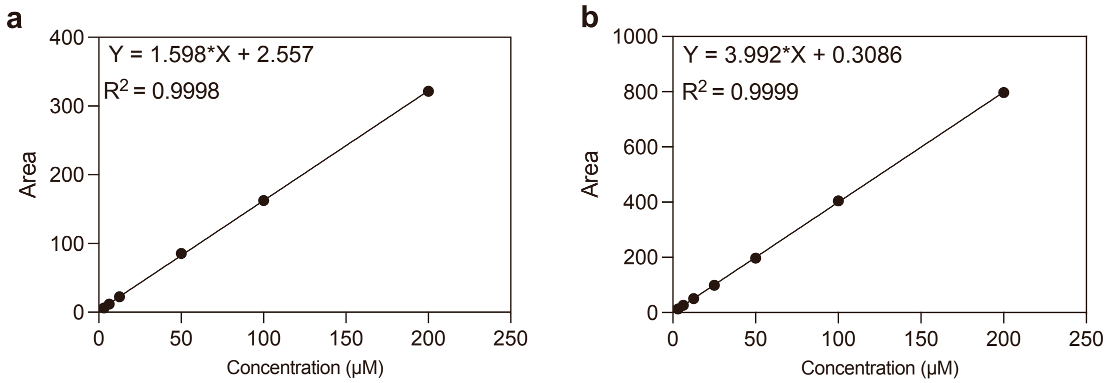

Figure S7. HPLC standard curves of different drugs. (a) HPLC standard curve used for Opto-PROTAC. (b) HPLC standard curve used for PROTAC.

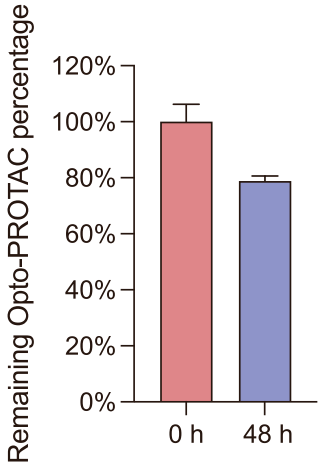

Figure S8. Stability analysis of Opto-PROTAC in the dark. Opto-PROTAC was dissolved in PBS solution at the final concentration of 100 μM and kept at 37 °C in the dark for 48 h. HPLC was utilized to analyze the concentration of the remaining Opto-PROTAC in the solution.
